# Ralstonia pickettii: Two Cases of Bacteremia in a Moroccan Pediatric Intensive Care Unit

**DOI:** 10.7759/cureus.82189

**Published:** 2025-04-13

**Authors:** Ibtissam Nabih, Youssef Idbaha, Ouissal Aissaoui

**Affiliations:** 1 Anesthesia and Critical Care, Ibn Rochd University Hospital, Hassan II University of Casablanca, Casablanca, MAR

**Keywords:** antibiotic resistance, bloodstream infection, nosocomial infection, pediatric intensive care, ralstonia pickettii

## Abstract

*Ralstonia pickettii *is an emerging opportunistic pathogen in hospital settings, particularly affecting immunocompromised patients. Its ability to persist in aqueous solutions and colonize medical devices contributes to nosocomial outbreaks, complicating infection prevention and management strategies.

We report two cases of *R. pickettii *bloodstream infections in the pediatric intensive care unit of a Moroccan university hospital. The first case involved an 11-month-old infant admitted with febrile respiratory distress. Despite initial treatment with imipenem and amikacin, *R. pickettii* bacteremia was diagnosed on day 30, requiring the addition of colistin and tigecycline. The clinical course was unfavorable, leading to death on day 40. The second case concerned a four-year-old child hospitalized for febrile status epilepticus. On day 10, a polymicrobial nosocomial infection involving *Pseudomonas aeruginosa*, *Acinetobacter baumannii*, and *R. pickettii *was identified, prompting treatment with colistin, tigecycline, and voriconazole. Despite therapeutic interventions, the patient succumbed to the infection on day 28.

The emergence of *R. pickettii *as a nosocomial pathogen necessitates reinforced infection control measures, improved diagnostic capabilities, and the development of tailored therapeutic protocols. Strict adherence to aseptic procedures and continuous hospital surveillance are essential to prevent the spread of this opportunistic bacterium.

## Introduction

Ralstonia pickettii, a non-fermenting Gram-negative bacillus, is primarily found in soil and water, where it demonstrates remarkable survival capacity in nutrient-poor environments due to its adaptability [1]. Long regarded as a harmless environmental organism, it is now recognized as an opportunistic pathogen, particularly concerning in hospital settings. Its ability to persist in aqueous solutions, including intravenous fluids and medical devices, has led to several outbreaks of nosocomial infections, drawing attention to its role in public health [1].

Infections caused by R. pickettii have been predominantly reported in immunocompromised individuals, including patients in intensive care units, organ transplant recipients, and those with chronic illnesses [2,3]. These infections are often associated with the use of invasive medical devices, such as catheters and infusion lines, which facilitate colonization and systemic infection [1]. In our context, as a developing country with limited resources, identifying R. pickettii is particularly challenging due to its slow growth on standard culture media and its potential to be misidentified as other Gram-negative bacilli. Furthermore, its intrinsic resistance to several antibiotics complicates its management even further [3]. We report here two cases of R. pickettii bacteremia identified in the pediatric intensive care unit of a tertiary care university hospital in Morocco.

## Case presentation

These two cases occurred in the pediatric intensive care unit of a university hospital in Morocco. The unit receives critically ill children from across the region, and often faces limited access to rapid diagnostic tools.

Case one

An 11-month-old infant with no significant medical history was admitted for febrile respiratory distress. On admission, clinical examination revealed a conscious child with a respiratory rate of 42 breaths per minute and an oxygen saturation (SpO₂) of 85% on room air, which improved to 92% under high-flow oxygen therapy (10 L/min). Heart rate was 90 beats per minute, blood pressure of 85/50 mmHg, and body temperature was 39.5°C. Signs of respiratory distress were present, including nasal flaring, subcostal and intercostal retractions, and tachypnea. Bilateral coarse crackles were noted on pulmonary auscultation. At admission, standard investigations including full blood count, renal and liver function tests, random blood sugar, chest X-ray, and urine analysis were performed as part of our initial assessment protocol.

Laboratory tests revealed leukocytosis at 22,120/mm³ and an elevated C-reactive protein (CRP) level of 217 mg/L. Treatment with imipenem (20 mg/kg every eight hours) for 10 days and amikacin (15 mg/kg/day) for three days was initiated at admission. Due to worsening respiratory and neurological symptoms, the patient required intubation on the second day of hospitalization.

By day 30, blood cultures identified *R. pickettii*, indicative of a nosocomial infection. Blood samples were collected under strict aseptic conditions by trained personnel, including skin disinfection with chlorhexidine, the use of sterile technique, and duplicate sampling from peripheral sites. Follow-up laboratory findings showed leukocytosis at 28,200/mm³ and a CRP level of 350 mg/L. Antibiotic therapy was adjusted to include colistin, tigecycline, and vancomycin. Unfortunately, the patient succumbed on the 40th day of hospitalization.

Case two

A four-year-old child, with no notable medical history, was admitted for febrile status epilepticus. On admission, the child was unconscious, with a Glasgow coma scale score of 9/15, assessed using the pediatric adaptation of the scale, an SpO₂ of 90%, a respiratory rate of 34 breaths per minute, a blood pressure of 100/60 mmHg, a heart rate of 126 beats per minute, and a temperature of 38.5°C. Bilateral coarse crackles were heard on pulmonary auscultation.

A brain CT scan revealed no abnormalities, and a lumbar puncture showed a cerebrospinal fluid (CSF) protein level of 0.37 g/L, a glucose level of 0.85 g/L, fewer than 3 white cells/mm³, and sterile cultures. Urinalysis (examen cyto-bacteriologique des urines (ECBU)) was also sterile. Blood tests showed leukocytosis at 21,360/mm³ and a CRP level of 10.7 mg/L. Given the neurological condition, the patient was intubated and treated with meningitis-dose ceftriaxone and acyclovir.

On day 10, a nosocomial infection was suspected due to persistent fever (39°C), leukocytosis at 25,340/mm³, and a CRP level of 281 mg/L. Bronchial samples revealed *Pseudomonas aeruginosa* and *Acinetobacter baumannii* resistant to imipenem, while blood cultures identified *R. pickettii.* Antibiotic therapy was adjusted to include tigecycline (1.2 mg/kg every 12 hours), colistin (1,000,000 IU/kg/day), and voriconazole (4 mg/kg every 12 hours). Despite these measures, the patient died on the 28th day of hospitalization. 

Table 1 shows a summary of the two cases. All samples were processed in the microbiology laboratory according to standard protocols, with identification and susceptibility testing performed using automated systems and following international quality guidelines.

**Table 1:** Summary of clinical cases *NR: Normal range, CRP: C-reactive protein

Case No.	Age	Sex	Reason for admission	Total leukocyte count (/mm³) (*NR: 6000 - 12000)		CRP level (mg/L) (*NR: < 5 )		Outcome
				Day 1	At the time of aggravation	Day 1	At the time of aggravation	
1	11 months	M	Febrile respiratory distress	22120	28200	217.0	350	Death on day 40
2	Four years	M	Febrile status epilepticus	21360	25340	10.7	281	Death on day 28

## Discussion

R*alstonia pickettii* is a Gram-negative opportunistic bacillus commonly found in aqueous and hospital environments [2]. Its ability to survive in nutrient-poor conditions and form biofilms on medical devices makes it a significant agent of nosocomial infections, particularly bacteremia and respiratory infections [2]. Its adaptive resistance to multiple antibiotics further complicates management, requiring a rigorous approach [2]. The two cases presented highlight the potential severity of nosocomial infections caused by R. pickettii in pediatric intensive care units. This pathogen, although rare, is capable of causing severe bacteremia in vulnerable patients.

This report illustrates the severe clinical course that can result from nosocomial *R. pickettii *infection in a previously healthy infant. Despite broad-spectrum empirical therapy and early respiratory support, the patient's condition worsened, leading to prolonged hospitalization and invasive interventions. In case one, the late identification of *R. pickettii* on day 30 strongly suggests a hospital-acquired infection, likely associated with the use of medical devices. Literature reports indicate that *R. pickettii*, like other Gram-negative opportunistic pathogens such as *P. aeruginosa* and *A. baumannii*, is capable of forming biofilms on the surfaces of medical devices, complicating eradication and promoting persistent infections [4]. Biofilms are structured communities of microorganisms adhering to a surface and surrounded by a protective extracellular matrix. This structure confers increased resistance to antimicrobial agents and host immune defenses [5].

The formation of biofilms by *R. pickettii* has been particularly observed in nutrient-poor environments, such as high-purity water systems used in pharmaceutical industries and hospitals [6]. A study by Adley et al. demonstrated that *R. pickettii* can colonize the internal surfaces of plastic pipes in such systems, forming biofilms that are difficult to eliminate. This capability is attributed to the production of signaling molecules, such as homoserine lactones (HSL), which play a key role in interbacterial communication and biofilm formation [6].

The presence of biofilms on medical devices such as catheters, probes, and implants poses a significant health risk for patients. Bacteria within biofilms exhibit increased tolerance to standard antimicrobial treatments, making infections difficult to treat and increasing the risk of complications [7]. Moreover, biofilms can act as reservoirs for bacterial dissemination throughout the body, leading to systemic infections [5].

To prevent the formation of *R. pickettii *biofilms on medical devices, it is essential to implement rigorous cleaning and sterilization protocols [8]. The use of materials resistant to bacterial adhesion and the development of antimicrobial coatings on devices can also reduce the risk of colonization [5]. Specific testing methods, such as the drip-flow biofilm reactor, allow the evaluation of prevention strategies under conditions simulating the real use of medical devices [5].

The second case involves a four-year-old child who developed a polymicrobial nosocomial infection by day 10 of hospitalization, including *R. pickettii*, *P. aeruginosa*, and *A. baumannii*, all resistant to imipenem. This illustrates the complexity of managing multidrug-resistant infections in pediatric intensive care settings. The limited data on *R. pickettii *susceptibility and its intrinsic resistance profile further narrowed therapeutic options, leading to the use of colistin and tigecycline.

The detection of *R. pickettii* is often delayed due to its slow growth on standard culture media and its potential misidentification as other Gram-negative bacilli [9]. The use of molecular diagnostic techniques, such as polymerase chain reaction (PCR), allows for rapid and precise identification of *R. pickettii*. These methods detect specific bacterial DNA, reducing the diagnostic delay compared to traditional cultures [10].

Strict adherence to aseptic protocols during the handling of medical devices and regular monitoring of potential contamination sources are essential to reduce the risk of *R. pickettii* infections [11]. Porto et al. reported in a prospective study that the incidence of nosocomial infections in pediatric intensive care can reach 22.1% of admissions, emphasizing the importance of rigorous preventive measures [12].

Given the multidrug resistance of *R. pickettii*, it is crucial to develop therapeutic protocols based on updated data [9]. The judicious use of antibiotics, avoiding inappropriate empirical treatments, is essential to limit the emergence of further resistance. Girlich et al. identified a chromosomal oxacillinase, OXA-60, in *R. pickettii*, capable of hydrolyzing imipenem [13]. This enzyme, expressed only after induction by β-lactams, confers increased resistance to carbapenems [14]. Thus, the inappropriate use of these antibiotics may induce the expression of such β-lactamases, reducing their efficacy and promoting the spread of resistant strains. It is therefore essential to base antibiotic choices on precise microbiological data and to avoid the empirical use of carbapenems without a clear indication.

Recent case reports highlight the emergence of *R. pickettii *as a nosocomial pathogen, particularly in immunocompromised patients. Nasir et al. reported three cases of *R. pickettii *bacteremia in a tertiary hospital, involving patients with complex medical histories and prolonged stays in intensive care units [2]. Demirdag et al. described an outbreak of R. pickettii bloodstream infections in pediatric leukemia patients, attributed to the use of contaminated solutions [15]. These cases underscore the need for heightened vigilance and stringent infection control measures to prevent the spread of this opportunistic pathogen in healthcare settings.

## Conclusions

The two cases presented highlight the severity of nosocomial infections caused by R. pickettii in pediatric intensive care units. The bacterium’s ability to form biofilms on medical devices and its intrinsic resistance to various antibiotics complicate both diagnosis and treatment. In our context, the lack of adequate healthcare infrastructure and advanced diagnostic techniques further exacerbates the challenges in detecting and managing this pathogen. These observations underscore the importance of strengthening infection prevention measures, improving diagnostic capabilities, and developing tailored therapeutic protocols to better address this opportunistic pathogen.
